# Allogeneic hematopoietic cell transplantation provides effective salvage despite refractory disease or failed prior autologous transplant in angioimmunoblastic T-cell lymphoma: a CIBMTR analysis

**DOI:** 10.1186/s13045-018-0696-z

**Published:** 2019-01-10

**Authors:** Narendranath Epperla, Kwang W. Ahn, Carlos Litovich, Sairah Ahmed, Minoo Battiwalla, Jonathon B. Cohen, Parastoo Dahi, Nosha Farhadfar, Umar Farooq, Cesar O. Freytes, Nilanjan Ghosh, Bradley Haverkos, Alex Herrera, Mark Hertzberg, Gerhard Hildebrandt, David Inwards, Mohamed A. Kharfan-Dabaja, Farhad Khimani, Hillard Lazarus, Aleksandr Lazaryan, Lazaros Lekakis, Hemant Murthy, Sunita Nathan, Taiga Nishihori, Attaphol Pawarode, Tim Prestidge, Praveen Ramakrishnan, Andrew R. Rezvani, Rizwan Romee, Nirav N. Shah, Ana Sureda, Timothy S. Fenske, Mehdi Hamadani

**Affiliations:** 1 Division of Hematology, Department of Medicine, The James Cancer Hospital and Solove Research Institute, The Ohio State University, 460 W 10th Ave, Columbus, OH 43210 USA; 2 Center for International Blood and Marrow Transplant Research, Department of Medicine, Medical College of Wisconsin, 9200 W. Wisconsin Avenue, Suite C5500, 8701 W. Watertown Plank Rd, Milwaukee, WI 53226 USA; 3 M.D. Anderson Cancer Center, 1515 Holcombe Boulevard, Houston, TX 77030 USA; 4 Sarah Cannon BMT Program, 2400 Patterson St. Suite 215, Nashville, TN 37206 USA; 5 Winship Cancer Institute, Emory University School of Medicine, 1365-C Clifton Road NE, Atlanta, GA 30322 USA; 6 Memorial Sloan Kettering Cancer Center, 1275 York Ave., New York, NY 10065 USA; 7 Shands Healthcare and University of Florida, PO Box 100278, Gainesville, FL 32610 USA; 8 University of Iowa Hospitals and Clinics, 200 Hawkins Drive C332 GH, Iowa City, IA 52242 USA; 9 Texas Transplant Institute, 4410 Medical Drive Suite 410, San Antonio, TX 78229 USA; 10 Levine Cancer Institute, 1021 Morehead Medical Drive Suite 5300, Charlotte, NC 28204 USA; 11 University of Colorado Hospital, 1665 Aurora Court F-754, Aurora, CO 80045 USA; 12 City of Hope National Medical Center, 1500 E Duarte Rd, Duarte, CA 91010 USA; 13 Prince of Wales Hospital, SEALS Level 4 Campus Building, Barker Street, Randwick, NSW 2031 Australia; 14 University of Kentucky Chandler Medical Center, 800 Rose Street CC 301, Lexington, KY 40536 USA; 15 Mayo Clinic Rochester, 200 First Street SW, Rochester, MN 55902 USA; 16 Mayo Clinic, 4500 San Pablo Rd, Jacksonville, FL 32224 USA; 17 H. Lee Moffitt Cancer Center and Research Institute, 12902 Magnolia Drive, Tampa, FL 33612 USA; 18 Case Western Reserve University, 11100 Euclid Ave, Cleveland, OH 44106 USA; 19 Univeristy of Miami, 1475 NW 12th Ave, Miami, FL 33136 USA; 20 Division of Hematology/Oncology, University Florida College of Medicine, 12902 Magnolia Drive, Tampa, FL 33612 USA; 21 Rush University Medical Center, 849 North Franklin Street Unit 1503, Chicago, IL 60610 USA; 22 The University of Michigan, 322 E Liberty St. Unit 4, Ann Arbor, MI 48104 USA; 23 Starship Children’s Health, Level 7 Blood and Cancer Center Park Road, Grafton, Auckland, 1142 New Zealand; 24 UT Southwestern Medical Center – BMT Program, 7800C Stenton Ave. Apt. 210, Philadelphia, PA 19118 USA; 25 Stanford Health Care, 300 Pasteur Drive, Room H0101 MC 5623, Stanford, CA 94305 USA; 26 Dana Farber Cancer Institute - Adults, 450 Brookline Avenue, Boston, MA 02215 USA; 27 Division of Hematology and Oncology, Department of Medicine, Medical College of Wisconsin, 8701 Watertown Plank Rd. PO Box 26509, Milwaukee, WI 53226 USA; 28 Institut Català d’Oncologia - Hospital Duran I Reynals, Avda. Granvfa 199-203, 08908 Barcelona, Spain

**Keywords:** Angioimmunoblastic T-cell lymphoma, Allogeneic transplantation, GVL effects

## Abstract

**Background:**

There is a paucity of data on the role of allogeneic hematopoietic cell transplantation (allo-HCT) in patients with angioimmunoblastic T-cell lymphoma (AITL). Using the CIBMTR registry, we report here the outcomes of AITL patients undergoing an allo-HCT.

**Methods:**

We evaluated 249 adult AITL patients who received their first allo-HCT during 2000–2016.

**Results:**

The median patient age was 56 years (range = 21–77). Majority of the patients were Caucasians (86%), with a male predominance (60%). Graft-versus-host disease (GVHD) prophylaxis was predominantly calcineurin inhibitor-based approaches while the most common graft source was peripheral blood (97%). Median follow-up of survivors was 49 months (range = 4–170 months). The cumulative incidence of grade 2–4 and grade 3–4 acute GVHD at day 180 were 36% (95% CI = 30–42) and 12 (95% CI = 8–17), respectively. The cumulative incidence of chronic GVHD at 1 year was 49% (95%CI 43–56). The 1-year non-relapse mortality (NRM) was 19% (95% CI = 14–24), while the 4-year relapse/progression, progression-free survival (PFS), and overall survival (OS) were 21% (95% CI = 16–27), 49% (95% CI = 42–56), and 56% (95% CI = 49–63), respectively. On multivariate analysis, chemoresistant status at the time of allo-HCT was associated with a significantly higher risk for therapy failure (inverse of PFS) (RR = 1.73 95% CI = 1.08–2.77), while KPS < 90% was associated with a significantly higher risk of mortality (inverse of OS) (RR = 3.46 95% CI = 1.75–6.87).

**Conclusion:**

Our analysis shows that allo-HCT provides durable disease control even in AITL patients who failed a prior auto-HCT and in those subjects with refractory disease at the time of allografting.

**Electronic supplementary material:**

The online version of this article (10.1186/s13045-018-0696-z) contains supplementary material, which is available to authorized users.

## Background

Angioimmunoblastic T-cell lymphoma (AITL) represents a distinct clinicopathologic entity among the mature T- and NK-cell neoplasms, accounting for approximately 1–2% of all non-Hodgkin lymphomas (NHLs) [1, 2]. AITL patients typically present with advanced stage disease, diffuse lymphadenopathy, hepatosplenomegaly, systemic symptoms, and hypergammaglobulinemia [3]. The clinical course is aggressive and the disease generally carries a poor prognosis even when treated with intensive induction regimens [3]. Standard first-line therapy mostly consists of anthracycline-based regimens with or without etoposide, based on the age [2, 4–6]. With this approach, overall survival (OS) is a little over 30% at 5 years [7]. In an attempt to improve the outcomes, autologous hematopoietic cell transplantation (auto-HCT) consolidation has been applied in this patient population [8–10]. While durable disease control can be observed typically in patients in first complete remission (CR), the outcomes of AITL subjects in partial remission (PR), and in those with refractory disease or treated with ≥ 2 prior therapy lines, following auto-HCT are less encouraging [10].

Allogeneic HCT (allo-HCT) may result in a lower risk of relapse in part due to a *graft*-versus*-lymphoma* effect mediated by the alloreactive donor cells [11–13]. Several retrospective studies [11, 14–16] have reported excellent disease control with low rates of relapse and a 1-year non-relapse mortality (NRM) ranging from 8 to 25% with allo-HCT in AITL patients. However, these analyses were done mainly in peripheral T-cell lymphoma (PTCL) patients with AITL as a subgroup or reported only a small number of patients with AITL (range *N* = 9–45 patients; Additional file 1: Table S1). We report here a registry analysis, evaluating the outcomes of patients with AITL undergoing allo-HCT.

## Methods

### Data sources

The Center for International Blood and Marrow Transplant Research (CIBMTR) is a working group of more than 500 transplantation centers worldwide that contribute detailed data on HCT to a statistical center at the Medical College of Wisconsin (MCW). Participating centers are required to report all transplantations consecutively and compliance is monitored by on-site audits. Computerized checks for discrepancies, physicians’ review of submitted data, and on-site audits of participating centers ensure data quality. Observational studies conducted by the CIBMTR are performed in compliance with all applicable federal regulations pertaining to the protection of human research participants. The MCW and National Marrow Donor Program, Institutional Review Boards approved this study.

The CIBMTR collects data at two levels: transplant essential data (TED) and comprehensive report form (CRF) data. TED data includes disease type, age, gender, pre-HCT disease stage and chemotherapy-responsiveness, date of diagnosis, graft type, conditioning regimen, post-transplant disease progression and survival, development of a new malignancy, and cause of death. All CIBMTR centers contribute to TED data. More detailed disease and pre- and post-transplant clinical information is collected on a subset of registered patients selected for CRF data by a weighted randomization scheme. TED- and CRF-level data are collected pre-transplant, 100-days, and 6 months post-HCT and annually thereafter or until death. Data for the current analysis were retrieved from CIBMTR (TED and CRF) report forms.

### Patients

Included in this analysis are adult (≥ 18 years) patients with AITL, undergoing their first allo-HCT between 2000 and 2016. Eligible donors included either HLA-identical sibling donors or unrelated donors (URD) matched at the allele-level at HLA-A, -B, -C, and -DRB1 and graft sources included peripheral blood and bone marrow. Graft-versus-host disease (GVHD) prophylaxis included both calcineurin inhibitor (CNI) and non-CNI-based regimens. Recipients of alternative donor transplantation were excluded due to small numbers (haploidentical allografts, *n* = 8; mismatched unrelated donor, *n* = 22; cord blood grafts, *n* = 21).

### Definitions and study endpoints

The intensity of conditioning regimens was defined using consensus criteria [17]. Disease response at the time of HCT was determined using the International Working Group criteria in use during the era of this analysis [18].

The primary endpoint was OS; death from any cause was considered an event and surviving patients were censored at last contact. Secondary endpoints included cumulative incidence of acute GVHD, chronic GVHD, GVHD free, relapse-free survival (GRFS), NRM, progression/relapse, and progression-free survival (PFS). NRM was defined as death without evidence of lymphoma progression/relapse; relapse was considered a competing risk. Progression/relapse was defined as progressive lymphoma after HCT or lymphoma recurrence after a CR; NRM was considered a competing risk. For PFS, a patient was considered treatment failure at the time of progression/relapse or death from any cause. Patients alive without evidence of disease relapse or progression were censored at last follow-up. Acute GVHD [19] and chronic GVHD [20] were graded using standard criteria. Neutrophil recovery was defined as the first of three successive days with absolute neutrophil count (ANC) ≥ 500/μL after post-transplantation nadir. Platelet recovery was defined as achieving platelet counts ≥ 20,000/μL for at least 3 days, unsupported by transfusion. For neutrophil and platelet recovery, death without the event was considered a competing risk. The causes of death are reported in accordance to the methodology described previously [21].

### Statistical analysis

Probabilities of PFS and OS were calculated using the Kaplan–Meier estimates. Cumulative incidence of NRM, lymphoma progression/relapse, and GVHD were calculated to accommodate for competing risks. Associations among patient-, disease-, and transplantation-related variables and outcomes of interest were evaluated using Cox proportional hazards regression. A stepwise model-building approach was used to identify covariates that influenced outcomes. Covariates with a *p* < 0.05 were considered statistically significant. The proportional hazards assumption for Cox regression was tested by adding a time-dependent covariate for each risk factor and each outcome. If a variable violated the proportional hazards assumption, it was added as a time-varying covariate. Interactions between the main effect and significant covariates were examined and none were found. Results are expressed as relative risks (RR). The center effect was examined using the random effect score test [22] for OS, PFS, relapse, and NRM. The variables considered in multivariate analysis are shown in Additional file 1: Table S2 of the supplemental appendix. All statistical analyses were performed using SAS version 9.4 (SAS Institute Inc., Cary, NC).

## Results

### Baseline characteristics

A total of 249 patients met the inclusion criteria and were included in this analysis. The baseline patient-, disease-, and transplantation-related characteristics are shown in Table 1. The median patient age was 56 years (range = 21–77 years). Most of the patients were Caucasians (86%), with a male (60%) predominance. The majority had a chemosensitive disease at the time of allo-HCT (79%) and received a non-myeloablative/reduced intensity conditioning regimen (73%). Most common type of GVHD prophylaxis included CNI ± methotrexate-based regimens. The graft source used for allo-HCT was predominantly peripheral blood (97%). Pre-transplant (allo-HCT) donor/recipient cytomegalovirus status was available in 200 patients (81%) and the details are provided in Table 1. There was no center effect noted on the outcomes. Median follow-up of survivors was 49 months (range, 4–170 months).

**Table 1:** Baseline patient characteristics of patients with AITL receiving first allo-HCT reported to the CIBMTR from 2000 to 2016

Variable	*N* = 249 (%)
Median age at HCT, years (range)	56 (21–77)
Male gender	150 (60)
Race	
Caucasian	214 (86)
African American	5 (2)
Others^a^	17 (7)
Missing	13 (5)
Karnofsky performance score ≥ 90	119 (48)
< 90	113 (45)
Missing	17 (7)
HCT-CI	
0	46 (18)
1–2	53 (21)
≥ 3	84 (34)
Not available before 2007	55 (22)
Missing	11 (4)
Interval from diagnosis to HCT, months	
Median (range)	14 (3–118)
Median lines of therapy before HCT (range)	3 (1–5)
Remission status at HCT	
Complete remission	108 (43)
Partial remission	90 (36)
Chemorefractory	38 (15)
Untreated/unknown	13 (5)
History of prior autologous HCT	98 (39)
TBI in conditioning	83 (34)
ATG/alemtuzumab in conditioning^b^	59 (24)
Conditioning intensity^c^	
Myeloablative conditioning	66 (27)
Non-myeloablative/RIC	183 (73)
Graft source	
Bone marrow	8 (3)
Peripheral blood	241 (97)
Donor type	
HLA-identical sibling	140 (56)
Unrelated donor 8/8	109 (44)
Donor/recipient CMV status	
Both negative	72 (29)
Both positive	59 (24)
Either donor/recipient +	69 (28)
Missing	49 (19)
Graft-versus-host disease prophylaxis	
Calcineurin inhibitor + MTX ± others^d^ (except MMF)	119 (48)
Calcineurin inhibitor + MMF ± others^d^	76 (31)
Calcineurin inhibitor + others (except MMF)	40 (16)
Others^d^	10 (4)
Missing	4 (2)
Year of HCT	
2000–2006	47 (19)
2007–2011	82 (33)
2012–2016	120 (48)
Median follow-up of survivors (range), months	49 (4–170)

*ATG* antithymocyte globulin, CMV cytomegalovirus, *HCT* hematopoietic cell transplantation, *HCT-CI* HCT-Comorbidity index, *MMF* mycophenolate mofetil, *MTX* methotrexate, *TBI* total body irradiation, *RIC* reduced intensity conditioning

^a^Others: 13 Asian; 3 Hispanic or Latino; 1 race unspecified, non-Hispanic

^b^ATG/alemtuzumab—49 ATG alone; 10 alemtuzumab alone

^c^For details, refer to Additional file 1: Table S4

^d^For details, refer to Additional file 1: Table S5

### Hematopoietic recovery

On univariate analysis, the cumulative incidence of neutrophil engraftment at 1-year was 97% (95% CI 94–99). The 1-year cumulative incidence of platelet recovery (Table 2) was 91% (95% CI 87–94).

**Table 2:** Univariate Analysis

Outcomes	N Eval	Prob (95% CI)
Neutrophil engraftment	**236**	
1-year		97 (94–99)%
2-year		97 (94–99)%
Platelet recovery	**218**	
1-year		91 (87–94)%
2-year		91 (87–95)%
Acute GVHD (II-IV)	**239**	
180-day		36 (30–42)%
Acute GVHD (III-IV)	**229**	
180-day		12 (8–17)%
Chronic GVHD	**230**	
1-year		49 (43–56)%
2-year		58 (51–64)%
Extensive cGVHD	**230**	
1-year		39 (33–46)%
2-year		46 (39–53)%
GRFS	**230**	
1-year		35 (29–41)%
2-year		27 (21–33)%
NRM	**249**	
1-year		19 (14–24)%
2-year		25 (20–31)%
4-year		30 (24–36)%
Progression/relapse	**249**	
1-year		15 (11–20)%
2-year		19 (15–25)%
4-year		21 (16–27)%
PFS	**249**	
1-year		66 (60–72)%
2-year		56 (49–62)%
4-year		47 (41–54)%
Overall survival	**249**	
1-year		73 (68–79)%
2-year		63 (56–69)%
4-year		56 (49–63)%

*GVHD* graft-versus-host disease, *Prob* probability, *CI* confidence interval, *N* number, *NRM* non-relapse mortality, *PFS* progression-free survival, *GRFS* GVHD free, relapse-free survival

Probabilities of acute GVHD, chronic GVHD, treatment-related mortality and progression/relapse were calculated using the cumulative incidence estimate. Progression-free survival and overall survival was calculated using the Kaplan-Meier product limit estimate

Univariate analysis of alternative donor sources is shown in Additional file 1 Table S6

### Acute and chronic GVHD

On univariate analysis, the cumulative incidence of grade II–IV acute GVHD was 36% (95% CI 30–42) and grades III–IV acute GVHD was 12% (95% CI 8–17) at day 180 (Table 2). None of the tested covariates (Additional file 1: Table S2) affected the risk of the development of acute GVHD.

On univariate analysis, the cumulative incidence of chronic GVHD at 1-year (Table 2) was 49% (95% CI 43–56), while the cumulative incidence of extensive chronic GVHD at 1 year (Table 2) was 39% (95% CI 33–46). Multivariate analysis (Table 3) showed that patients who received anti-thymocyte globulin (ATG) or alemtuzumab had a significantly lower risk of chronic GVHD (RR = 0.58, 95% CI 0.36–0.93, *p* = 0.02) relative to those who did not receive ATG/alemtuzumab.

**Table 3:** Multivariate analysis results

	Number	RR	95% CI lower limit	95% CI upper limit	*P*-value	Overall *p* value
Chronic GVHD						
ATG/alemtuzumab						
No	174	1				*0.02*
Yes	55	0.58	0.36	0.93	*0.02*	
Progression/Relapse						
No significant covariates						
Non-relapse mortality						
No significant covariates						
Progression-free survival						
Disease status						
CR	108	1				*0.03*
PR	90	1.13	0.76	1.66	0.54	
Chemoresistant	38	1.73	1.08	2.77	*0.02*	
Missing/Untreated	13	0.43	0.15	1.20	0.11	
Overall survival						
Karnofsky performance score (≤ 6 months)^a^						
≥ 90%	119	1				*0.002*
< 90%	113	3.46	1.74	6.87	*0.0004*	
Missing	17	1.95	0.54	6.98	0.31	
Karnofsky performance score (> 6 months)^a^						
≥ 90%	106	1				0.28
< 90%	80	0.66	0.39	1.12	0.12	
Missing	14	0.73	0.29	1.86	0.51	

*GVHD* graft-versus-host disease, *CI* confidence interval, *ATG* anti-thymocyte globulin, *CR* complete remission, *PR* partial remission, *RR* relative risk

Variables tested in the Multivariate analysis are listed in Additional file 1 Table S2

^a^6-months was chosen as cut-off based on the maximum likelihood value in the Cox model

*p*-value <0.05 is considered significant

### Transplantation outcomes

On univariate analysis, the cumulative incidence of 1-year GRFS (Table 2) was 35% (95% CI 29–41).

**Fig. 1:**
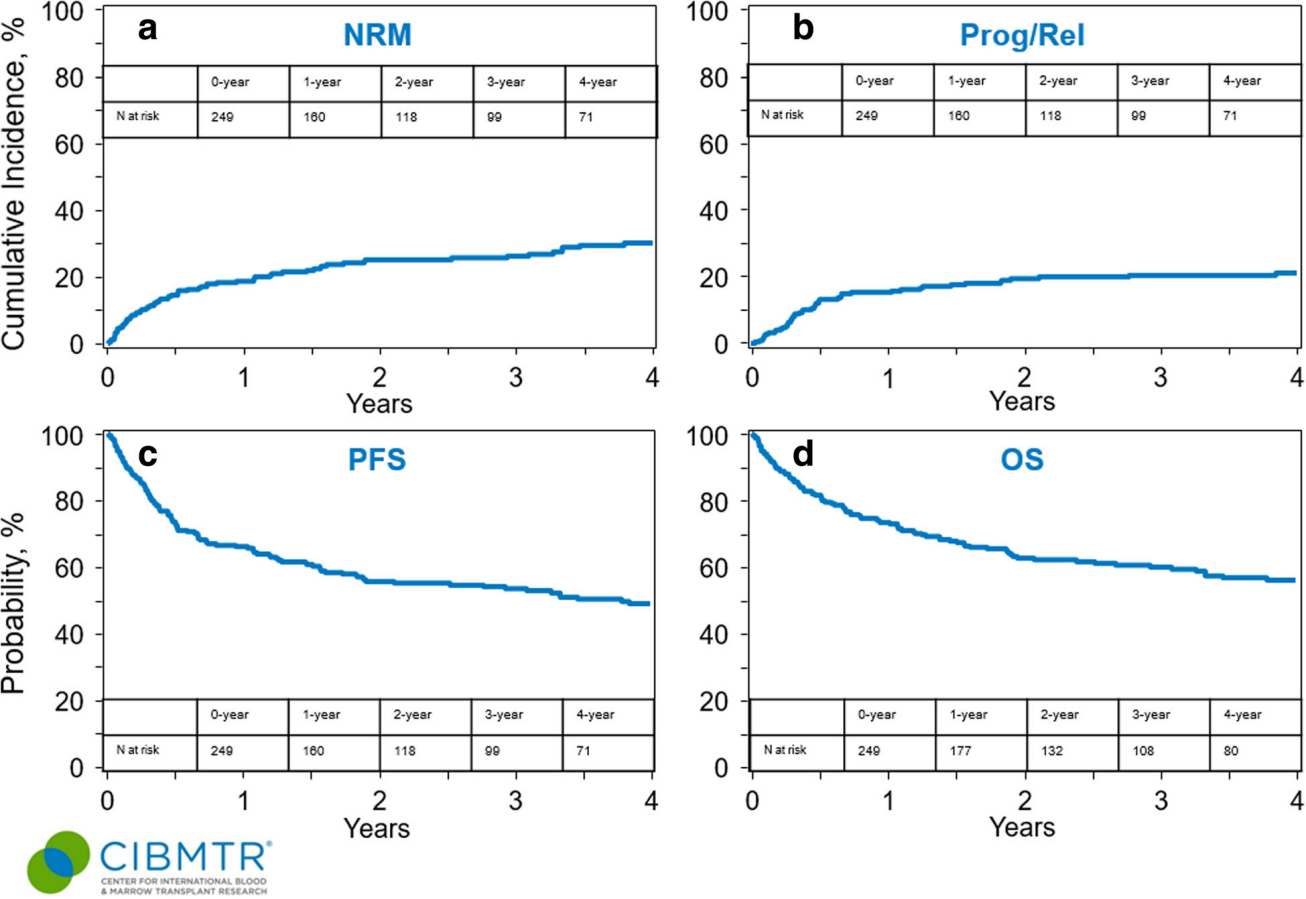
Outcomes of patients receiving first allo-HCT for AITL. **a** Cumulative incidence of non-relapse mortality. **b** Cumulative incidence of lymphoma progression/relapse. **c** Progression-free survival. **d** Overall survival

The 1-year NRM rate (Table 2) was 19% (95% CI 14–24) (Fig. 1a). On multivariate analysis, there were no significant covariates affecting the risk of NRM. The cumulative incidence of progression/relapse at 4 years (Table 2) was 21% (95% CI 16–27) (Fig. 1b). On multivariate analysis (Table 3), none of the covariates (Additional file 1: Table S1, including chronic GVHD assessed as a time-dependent variable) significantly affected the relapse risk.

The 4-year PFS and OS (Table 2) were 47% (95% CI 41–54) (Fig. 1c) and 56% (95% CI 49–63) (Fig. 1d), respectively. On multivariate analysis (Table 3), chemoresistant status at the time of allo-HCT significantly increased the risk for therapy failure (inverse of PFS) (RR = 1.73 95% CI = 1.08–2.77, *p* = 0.02), while KPS < 90% was associated with a significantly higher risk of mortality (inverse of OS) in the first 6-months post allo-HCT (RR = 3.46 95% CI = 1.74–6.87, *p* = 0.0004).

### Causes of death

At last follow-up, 45% (*n* = 112) of allo-HCT recipients had died (Additional file 1: Table S3). The most common cause of death was organ failure, 20% (*n* = 22) followed by recurrent/progressive disease, 19% (*n* = 21). GVHD was the cause of death in 17% (*n* = 19) and infectious complications accounted for death in 15% (*n* = 17) of patients. The other causes of death are listed in Additional file 1: Table S3.

### Impact of prior autograft and disease status

**Fig. 2:**
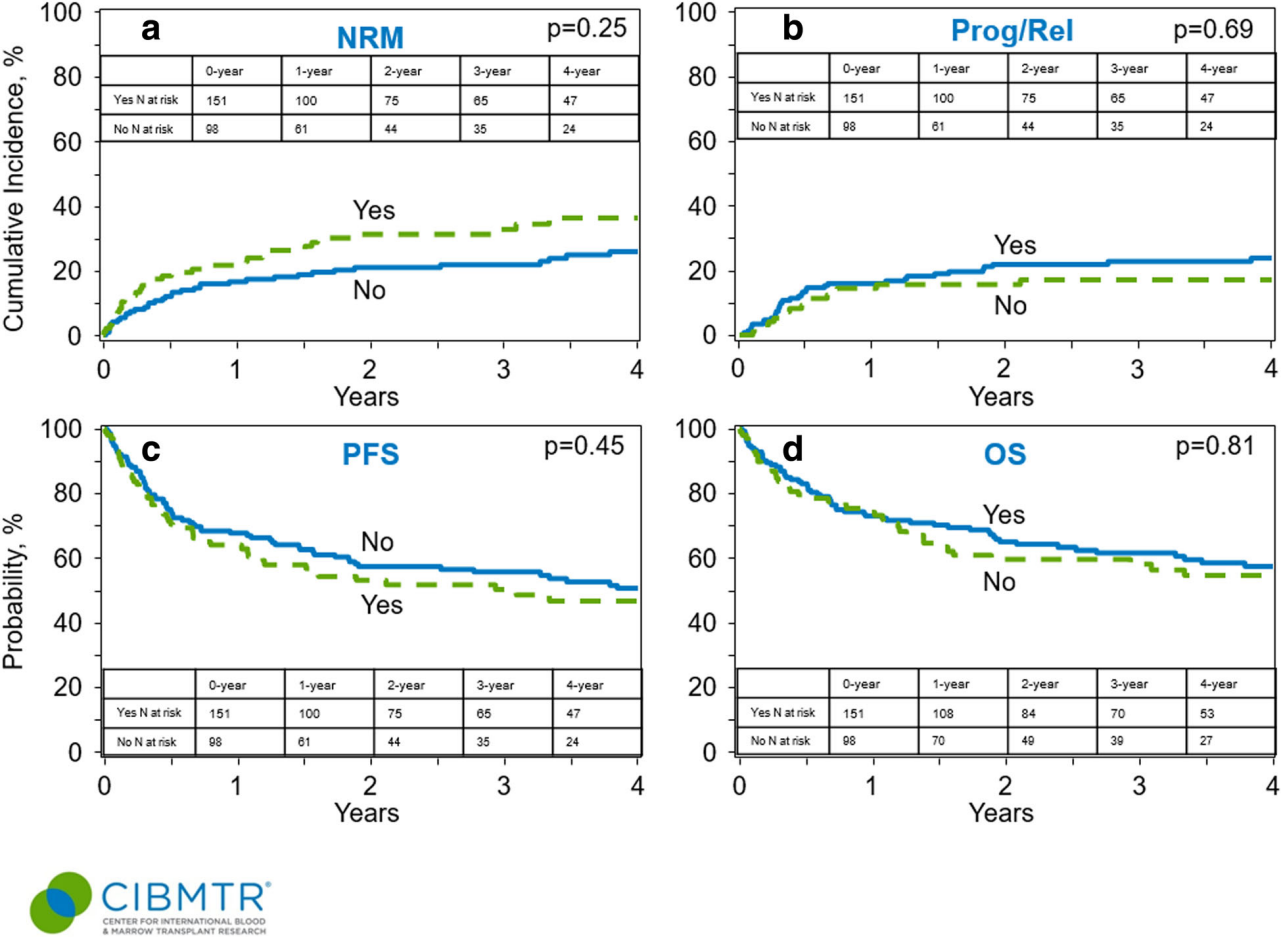
Outcomes of AITL patients based on the receipt of prior auto-HCT vs no prior auto-HCT. **a** Cumulative incidence of non-relapse mortality. **b** Cumulative incidence of lymphoma progression/relapse. **c** Progression-free survival. **d** Overall survival

Among the 249 patients who received first allo-HCT, 98 patients (39%) had received a prior auto-HCT. Univariate analysis looking at the impact of prior auto-HCT (no prior auto-HCT vs prior auto-HCT) on the outcomes showed no significant difference in the 1-year NRM (17% [95% CI 11–23] vs 22% [95% CI 14–30], *p* = 0.33), 4-year progression/relapse (24% [95% CI 17–31] vs 17% [95% CI 10–25], *p* = 0.21), PFS (50% [95% CI 42–59] vs 47% [95% CI 36–57], *p* = 0.60), or OS (57% [95% CI 49–65] vs 54% [95% CI 44–65], *p* = 0.70) (Table 4**,** Fig. 2).

**Table 4:** Comparative analysis of AITL patients who received prior auto-HCT vs no prior auto-HCT

Outcomes	No prior auto-HCT (*N* = 151)		Prior auto-HCT (*N* = 98)		*p* value
	*N*	Prob (95% CI)	*N*	Prob (95% CI)	
NRM	**151**		**98**		0.25
1-year		17 (11–23)%		22 (14–30)%	0.33
2-year		21 (15–28)%		31 (22–41)%	0.08
3-year		22 (16–29)%		33 (23–43)%	0.07
4-year		26 (19–34)%		36 (26–47)%	0.11
Progression/relapse	**151**		**98**		0.69
1-year		16 (11–22)%		15 (8–22)%	0.77
2-year		22 (15–29)%		16 (9–24)%	0.23
3-year		23 (16–30)%		17 (10–25)%	0.28
4-year		24 (17–31)%		17 (10–25)%	0.21
PFS	**151**		**98**		0.45
1-year		68 (60–75)%		64 (54–73)%	0.56
2-year		57 (49–65)%		53 (43–63)%	0.53
3-year		55 (47–64)%		50 (40–61)%	0.43
4-year		50 (42–59)%		47 (36–57)%	0.60
Overall survival	**151**		**98**		0.81
1-year		73 (65–80)%		74 (65–82)%	0.81
2-year		65 (57–72)%		59 (49–69)%	0.43
3-year		61 (53–69)%		58 (47–68)%	0.63
4-year		57 (49–65)%		54 (44–65)%	0.70

*Prob* probability, *CI* confidence interval, *N* number, *NRM* non-relapse mortality, *PFS* progression-free survival, *HCT* hematopoietic cell transplantation

**Fig. 3:**
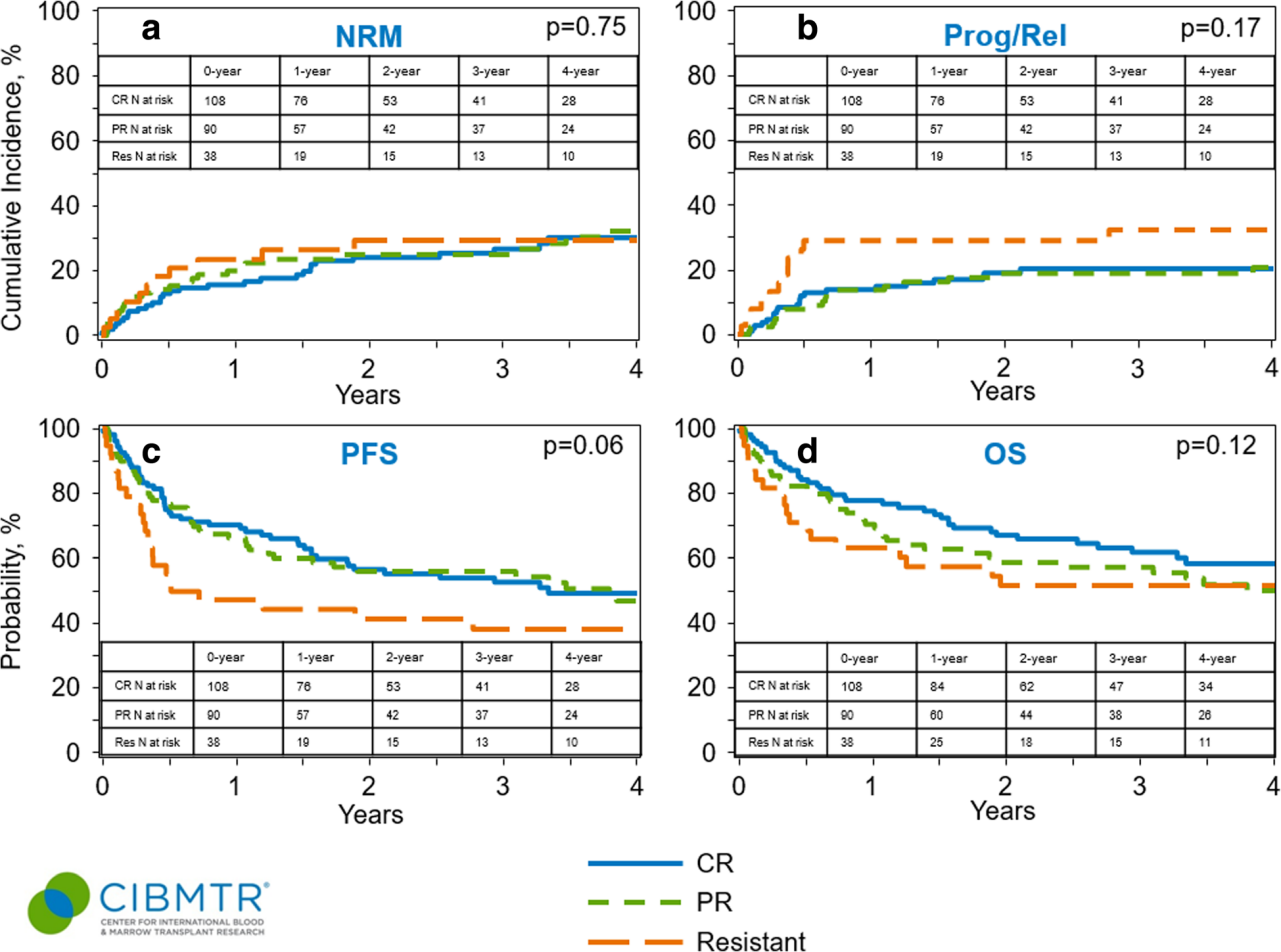
Outcomes of AITL patients based on the disease status at allo-HCT. **a** Cumulative incidence of non-relapse mortality. **b** Cumulative incidence of lymphoma progression/relapse. **c** Progression-free survival. **d** Overall survival

Among the 198 patients with chemosensitive disease at the time of allo-HCT, 33 patients (17%) were in CR1, while 75 patients (38%) were in CR > 1 and 90 patients (45%) were in PR. Univariate analysis looking at the effect of remission status at allo-HCT, CR1 vs CR > 1 vs PR vs refractory (Table 5), showed a 4-year PFS of 58% vs 45% vs 47% vs 38%, respectively, and a 4-year OS of 70% vs 54% vs 50% vs 52%, respectively. Among patients with chemorefractory AITL, the 1-year NRM was 24%, while the 4-year progression/relapse, PFS, and OS in patients with refractory AITL were 32%, 38%, and 52%, respectively. Figure 3 shows the disease outcomes for AITL patients based on the remission status at allo-HCT (CR vs PR vs chemoresistant).

**Table 5:** Comparative analysis of AITL patients based on the remission status at the time of allo-HCT

	CR1 (*N* = 33)		CR > 1 (*N* = 75)		PR (*N* = 90)		Refractory (*N* = 38)	
Outcomes	*N*	Prob (95% CI)	*N*	Prob (95% CI)	*N*	Prob (95% CI)	*N*	Prob (95% CI)
NRM	**33**		**75**		**90**		**38**	
1-year		6 (1–17)%		20 (12–30)%		20 (13–29)%		24 (12–38)%
2-year		13 (4–26)%		29 (19–40)%		25 (17–35)%		30 (16–45)%
3-year		17 (6–32)%		31 (21–43)%		25 (17–35)%		30 (16–45)%
4-year		17 (6–32)%		36 (25–49)%		33 (22–44)%		30 (16–45)%
Progression/ relapse	**33**		**75**		**90**		**38**	
1-year		15 (5–29)%		13 (7–22)%		14 (7–21)%		29 (16–44)%
2-year		25 (12–41)%		16 (9–26)%		19 (11–28)%		29 (16–44)%
3-year		25 (12–41)%		18 (10–28)%		19 (11–28)%		32 (18–48)%
4-year		25 (12–41)%		18 (10–28)%		21 (12–30)%		32 (18–48)%
PFS	**33**		**75**		**90**		**38**	
1-year		79 (63–91)%		67 (56–77)%		66 (56–76)%		47 (32–63)%
2-year		62 (45–78)%		54 (43–66)%		56 (45–66)%		41 (26–57)%
3-year		58 (41–75)%		50 (38–62)%		56 (45–66)%		38 (23–54)%
4-year		58 (41–75)%		45 (33–58)%		47 (36–58)%		38 (23–54)%
Overall survival	**33**		**75**		**90**		**38**	
1-year		88 (75–97)%		73 (63–83)%		71 (61–80)%		63 (47–78)%
2-year		78 (62–90)%		62 (51–73)%		59 (48–69)%		52 (36–67)%
3-year		70 (52–85)%		58 (46–70)%		57 (47–68)%		52 (36–67)%
4-year		70 (52–85)%		54 (41–66)%		50 (39–62)%		52 (36–67)%

*CR* complete response, *PR* partial response, *Prob* probability, *CI* confidence interval, *N* number, *NRM* non-relapse mortality, *PFS* progression-free survival

## Discussion

Prospective studies evaluating the outcomes of allo-HCT exclusively in AITL have not been performed given an overall rarity of this PTCL subtype. Here, we performed a registry analysis of AITL patients receiving first allo-HCT and made several important observations. First, allo-HCT provided durable disease control in patients with AITL as evidenced by 4-year PFS of 47%. Second, the risk of relapse tended to plateau at 2-year post allo-HCT. Lastly, allo-HCT provided durable disease control even in patients with a failed prior auto-HCT and those subjects with refractory disease at the time of allografting.

Auto-HCT has been previously studied as a consolidation modality for patients with AITL in first CR and beyond. While auto-HCT can provide durable disease control in AITL subjects in CR1, the outcomes of patients not in CR, or those with heavily pretreated disease are not optimal [10]. In addition, despite low transplant-related mortality, the risk of relapse following autografting remains high (1- and 2-year relapse risk is 40% and 51%, respectively) [10]. In contrast, allo-HCT provides excellent survival outcomes for patients with AITL with a lower risk of relapse. Additional file 1: Table S1 summarizes the retrospective studies (*n* ≥ 9) that have looked at the role of allo-HCT in AITL [11, 14–16]. The current study is the largest registry validation of these results showing durable responses in patients with AITL following allo-HCT. Though previous studies included patients with prior auto-HCT failure and chemorefractory state, the data are limited by very small patient numbers (for example, the previously published study with a large number of AITL patients [*n* = 45] included 15 patients with prior auto-HCT failure and 18 patients with chemorefractory disease at allo-HCT) [14] limiting the ability to draw meaningful conclusions. Considering the fact that ASBMT Clinical Practice Recommendation Panel [23] endorses the use of auto-HCT in AITL patients in CR1/PR1, and the high rates of disease relapse in patients receiving high-dose therapy, addressing the role of a subsequent allo-HCT is a clinically important question. In the current analysis, we did not observe any statistically significant differences in outcomes for patients who had prior auto-HCT vs no prior auto-HCT. Our results support the curative potential of allo-HCT in high-risk AITL patients who have failed a prior auto-HCT.

Limited data are published on the role of allo-HCT in refractory AITL. Registry data from the European Society for Blood and Marrow Transplantation (EBMT) identified chemorefractory disease as a predictor of inferior outcomes but included only 18 refractory AITL patients [14]. In the current analysis, the 4-year PFS and OS of chemorefractory patients was 38% and 52% respectively, which supports the use of allografting in this ultra-high-risk subset of patients (who otherwise are fit to undergo allo-HCT). In our study, we did not find a relationship between chronic GVHD and relapse rate in contrast to the previously reported data [14]. The retrospective nature of the registry data does not permit us to analyze the optimal timing of allo-HCT. While the outcomes of CR1 patients in the current study were favorable (4-year PFS and OS 58% and 70%), prior studies have also suggested very encouraging outcomes of AITL patients undergoing auto-HCT in CR1 [10, 24].

AITL is a challenging diagnosis with roughly only 80% concordance even among expert pathologists with access to archival tissue [3, 7]. One of the limitations of the current study is the lack of central pathology review of archival tissue for all patients. The current study included cases as diagnosed by the pathologists at the respective institutions. Of note, disease histology is one of the critical fields CIBMTR examines during its onsite transplant center audits (where diagnosis reported to CIBMTR is audited relative to the pathology records available at the reporting center). In recent CIBMTR studies involving rare T-cell histologies, > 95% concordance was seen between center-reported diagnosis and central review of pathology reports [25, 26]. We acknowledge that this analysis is not a substitute of central review of archival tissue by expert pathologists. At the same time, it is important to note that the majority of prospective clinical trials enrolling AITL subjects accept the patients based on the pathology reports at the participating sites, without a mandatory central review of archival tissue. In addition, the CIBMTR registry does not capture post-relapse salvage therapy, thereby limiting the ability to assess the post allo-HCT relapse survival.

## Conclusions

With a better understanding of the biology and development of prognostic tools, there has been a major effort to study novel drug combinations and immunotherapy agents (including checkpoint inhibitors and chimeric antigen receptor T-cell [CAR-T] therapy) in patients with NHL. Brentuximab vedotin (anti-CD30 antibody-drug conjugate) is being studied in combination with chemotherapy in the frontline setting in PTCL patients (ECHELON 2 trial, NCT 01777152). The final results are eagerly awaited to assess the impact of CD30-directed therapies in the subset of AITL patients. While the data on CAR-T cell therapy for B-cell NHL (mainly diffuse large B-cell lymphoma) in the relapsed/refractory setting is impressive [27], similar constructs in T-cell NHL have not been translated to the bedside. Our results suggest that allo-HCT offers the potential for cure in AITL patients including those with otherwise chemo-refractory disease. In the foreseeable future, allo-HCT is likely to remain an important therapeutic option for AITL patients.

## Additional file

Additional file 1: **Table S1.** Outcomes of patients with AITL who underwent allogeneic HCT. **Table S2.** Variables tested in Cox proportional hazards regression models. **Table S3.** Causes of Death. **Table S4.** Conditioning Intensity. **Table S5.** Details of GVHD prophylaxis regimens. **Table S6.** Univariate outcomes of AITL patients receiving alternative donor sources. (DOCX 29 kb)

## Abbreviations

AITL: Angioimmunoblastic T-cell lymphoma
Allo-HCT: Allogeneic hematopoietic cell transplantation
CNI: Calcineurin inhibitor
CR: Complete remission
CRF: Comprehensive Report Form
GRFS: GVHD free, relapse-free survival
GVHD: Graft-versus-host disease
NRM: Non-relapse mortality
OS: Overall survival
PFS: Progression-free survival
PR: Partial remission
RR: Relative risk
TED: Transplant essential data
