# Supplementary material for: Allogeneic hematopoietic cell transplantation provides effective salvage despite refractory disease or failed prior autologous transplant in angioimmunoblastic T-cell lymphoma: a CIBMTR analysis
Source: J Hematol Oncol. 2019 Jan 10;12:6. doi: 10.1186/s13045-018-0696-z (PMC6329157; doi:10.1186/s13045-018-0696-z)
Supplement: Supplementary file 1 — Table S1. Outcomes of patients with AITL who underwent allogeneic HCT. Table S2. Variables tested in Cox proportional hazards regression models. Table S3. Causes of Death. Table S4. Conditioning Intensity. Table S5. Details of GVHD prophylaxis regimens. Table S6. Univariate outcomes of AITL patients receiving alternative donor sources. (DOCX 29 kb) [file 13045_2018_696_MOESM1_ESM.docx]

**Additional file**

**Table of Contents 1**

**Summary of Published data:** Table S1 2

**Variables tested in MVA:** Table S2 3

**Causes of death:** Table S3 4

**Details of Conditioning Intensity:** Table S4 5

**Details of GVHD prophylaxis:** Table S5 6

**Outcomes for alternative donor sources:** Table S6 7

**Table S1: Outcomes of patients with AITL who underwent allogeneic HCT**

| **Authors**  **(year)** | **Study type** | **N** | **Preparative regimens** | **aGVHD**  **(2-4)** | **cGVHD (1 yr)** | **NRM** | **Prog/**  **Relapse** | **PFS** | **OS** |
| --- | --- | --- | --- | --- | --- | --- | --- | --- | --- |
| Le Gouill  (2008) | Retrosp study | 11 | MAC=55% NMA=45% | - | - | 1 yr: 9% | 5 yr: 9% | 5 yr: 80%^1^ | 5 yr: 80% |
| Kyriakou C (2009) | Retrosp study | 45 | MAC=56% RIC=44% | 29% | 52% | 1 yr: 25%  3 yr: 27% | 1 yr: 13%  3 yr: 20% | 1 yr: 62%  3 yr: 53% | 1 yr: 66%  3 yr: 64% |
| Dodero (2012) | Retrosp study | 9 | MAC=64%^2^ RIC=36% | 21%^2^ | 17%^2^ | 5 yr: 12%^2^ | 5 yr: 49%^2^ | 5 yr: 44% | 5 yr: 66% |
| Smith SM (2013) | Retrosp study | 12 | - | 8% | 27% | 1 yr: 8%  3 yr: 8% | 1 yr: 25%  3 yr: 25% | 1 yr: 67%  3 yr: 67% | 1 yr: 92%  3 yr: 83% |

Abbreviations: Retrosp=retrospective; aGVHD=acute graft versus host disease; cGVHD= chronic graft versus host disease;

NRM=non-relapse mortality; Prog=progression; PFS=progression-free survival; OS= overall survival; MAC=myeloablative

conditioning; RIC=reduced-intensity conditioning.

^1^Indicates Event-free survival
^2^Depicts the outcome of the entire PTCL cohort (n=52)

**Table S2: Variables tested in Cox proportional hazards regression models**

**Patient-related:**

- Age at transplant, continuous and by age groups (decades)
- Patient sex: male vs. female
- Race: Caucasians vs. others vs missing
- Karnofsky performance status at transplant: 90-100% vs. <90% vs. missing
- Sorror Co-morbidity index : 0 vs. 1-2 vs. 3 or more

**Disease-related:**

- Time from diagnosis to HCT: ≥1 year vs. <1 year
- Chemosensitivity at allo-HCT: CR vs. PR vs. chemoresistant vs. untreated/missing
- Prior autologous transplant: No vs. Yes

**Transplant-related:**

- Year of transplant: 2000-2006 vs 2007-2011 vs 2012-2016
- ATG/alemtuzumab use in conditioning: No vs. Yes vs. missing
- Donor type: HLA-identical sibling vs. URD
- Conditioning intensity: Myeloablative (MAC) vs. reduced-intensity conditioning (RIC)
- GVHD prophylaxis: calcineurin inhibitors + MTX ± others (excluding MMF) vs. calcineurin inhibitors + MMF ± others vs. calcineurin inhibitors + others (excluding MTX/MMF) vs. others vs. missing
- Donor/Recipient CMV status : -/+ vs. others
- cGVHD (as a time-dependent covariate in model of progression/relapse)

**Table S3: Causes of Death**

| **Cause of death** | **N (%)** |
| --- | --- |
| Number of deaths | 112 |
| Organ failure | 22 (20) |
| Primary disease | 21 (19) |
| Graft versus Host disease | 19 (17) |
| Infection | 17 (15) |
| Second malignancy | 5 (4) |
| Hemorrhage | 4 (3) |
| Acute Respiratory Distress Syndrome | 1 (<1) |
| Other causes^1^ | 2 (2) |
| Unknown | 21 (19) |

^1^Other cause: 1 TENS; 1 leukoencephalopathy.

**Table S4: Conditioning Intensity**

| **Conditioning regimen by intensity** | **N=249 (%)** |
| --- | --- |
| **Myeloablative** | **66 (27)** |
| Cy/TBI ±others^1^ | 33 (13) |
| Flu/Bu | 12 (5) |
| Bu/Cy | 7 (3) |
| Flu/Mel ± others^2^ | 6 (2) |
| TBI ±other(s)^3^ | 5 (2) |
| Other(s)^4^ | 2 (<1) |
| Missing | 1 (<1) |
| **Non-myeloablative/RIC** | **183 (73)** |
| Flu/Mel ± others^5^ | 63 (25) |
| Flu/Bu | 39 (16) |
| TBI/Flu | 22 (9) |
| Cy/Flu | 12 (5) |
| Cy/TBI/Flu ± others^6^ | 10 (4) |
| BEAM like | 9 (4) |
| TBI ± other(s)^7^ | 8 (3) |
| Flu ± Other^8^ | 5 (2) |
| TLI | 7 (3) |
| Other(s)^9^ | 7 (3) |
| Missing | 1 (<1) |

Abbreviations: Flu=fludarabine; Bu=busulfan; Cy=cyclophosphamide; Mel=melphalan; TBI=total body irradiation; BEAM=Carmustine, etoposide, cytarabine and melphalan; TLI=total lymphoid irradiation; RIC=reduced-intensity conditioning

**MAC:**
^1^Cy/TBI ± others: Cy/TBI alone=28; Cy/TBI/ATG=1; Cy/TBI/Etoposide=2; Cy/TBI/Flu/TT=2
^2^lu/Mel ± others: Flu/Mel alone=2; Flu/Mel/ATG/Nitro=1; Flu/Mel/ATG/TT=1; Flu/Mel/Mab=1; Flu/Mel/Nitro=1
^3^TBI ± others: TBI alone=4; TBI/Flu/TT=1
^4^Others: Bu alone=1; Mel/Etoposide=1

**NMA/RIC:**
^5^Flu/Mel ± others: Flu/Mel alone=46; Flu/Mel/ATG=9; Flu/Mel/ATG/Nitro=2; Flu/Mel/Mab=4; Flu/Mel/Nitro=1; Flu/Mel/This=1
^6^Cy/TBI/Flu ± others: Cy/TBI/Flu=6; Cy/TBI/Flu/TT=1; Cy/TBI/Flu/ATG=3
^7^TBI ± other(s): TBI alone=5; TBI/Pentostatin=3
^8^Flu ± Other: Flu alone=4; Flu/Bendamustine=1
^9^Other(s): Bu alone= 2; TBI/Mel=2; Cy/TBI/Etoposide=1; TBI/Etoposide=1; Cy/TBI=1

**Table S5: Details of GVHD prophylaxis regimens.**

| **Variable** | **N=249 (%)** |
| --- | --- |
| **CNI + MTX +/- others (except MMF)** | 119 (48) |
| CNI + MTX | 93 |
| CNI + MTX + Sirolimus | 20 |
| CNI + MTX + ATG | 2 |
| CNI + MTX + corticosteroids | 2 |
| CNI + MTX + ECP | 1 |
| CNI + MTX + monoclonal antibody | 1 |
| **CNI + MMF +/- others** | 76 (31) |
| CNI + MMF | 60 |
| CNI + MMF + MTX | 8 |
| CNI + MMF + ATG | 4 |
| CNI + MMF + Sirolimus | 3 |
| CNI + MMF + monoclonal antibody | 1 |
| **CNI+- others (except MMF)** | 40 (16) |
| CNI | 19 |
| CNI + Sirolimus | 17 |
| CNI + monoclonal antibody | 3 |
| CNI + corticosteroids | 1 |
| **Others** | 10 (4) |
| MTX | 1 |
| MMF | 1 |
| MTX+MMF | 1 |
| Post-Cy | 3 |
| Ex-vivo T-cell depletion | 4 |
| Missing | 4 (2) |

Abbreviations: CNI=Calcineurin inhibitor; MTX=methotrexate; MMF=mycophenolate mofetil; ATG=anti-thymocyte globulin; ECP=extracorporeal photopheresis; Cy=cyclophosphamide

**Table S6: Univariate outcomes of AITL patients receiving alternative donor sources**

|  | **Haploidentical donor**  **(N = 8)** | | **Mismatch unrelated donor (N = 22)** | | **Cord blood**  **(N = 21)** | |  |
| --- | --- | --- | --- | --- | --- | --- | --- |
| **Outcomes** | **N Eval** | **Prob (95% CI)** | **N Eval** | **Prob (95% CI)** | **N Eval** | **Prob (95% CI)** | **p-value** |
| **Chronic GVHD** | 7 |  | 21 |  | 19 |  | 0.03 |
| 1-year |  | 33 (5-72)% |  | 48 (27-69)% |  | 21 (6-42)% | 0.18 |
| 2-year |  | 33 (5-72)% |  | 65 (44-84)% |  | 28 (10-51)% | 0.04 |
| **NRM** | 8 |  | 22 |  | 21 |  | 0.14 |
| 1-year |  | 13 (0-42)% |  | 18 (5-37)% |  | 38 (19-59)% | 0.20 |
| 2-year |  | 13 (0-42)% |  | 18 (5-37)% |  | 38 (19-59)% | 0.20 |
| 3-year |  | 13 (0-42)% |  | 18 (5-37)% |  | 38 (19-59)% | 0.20 |
| 4-year |  | 13 (0-42)% |  | 18 (5-37)% |  | 38 (19-59)% | 0.20 |
| **Progression/relapse** | 8 |  | 22 |  | 21 |  | 0.29 |
| 1-year |  | 28 (3-63)% |  | 32 (14-52)% |  | 24 (9-44)% | 0.84 |
| 2-year |  | 28 (3-63)% |  | 37 (18-58)% |  | 24 (9-44)% | 0.64 |
| 3-year |  | 28 (3-63)% |  | 42 (22-63)% |  | 24 (9-44)% | 0.43 |
| 4-year |  | 28 (3-63)% |  | 42 (22-63)% |  | 24 (9-44)% | 0.43 |
| **PFS** | 8 |  | 22 |  | 21 |  | 0.33 |
| 1-year |  | 60 (25-90)% |  | 50 (30-70)% |  | 38 (19-59)% | 0.53 |
| 2-year |  | 60 (25-90)% |  | 45 (25-66)% |  | 38 (19-59)% | 0.58 |
| 3-year |  | 60 (25-90)% |  | 40 (21-61)% |  | 38 (19-59)% | 0.56 |
| 4-year |  | 60 (25-90)% |  | 40 (21-61)% |  | 38 (19-59)% | 0.56 |
| **Overall survival** | 8 |  | 22 |  | 21 |  | 0.55 |
| 1-year |  | 75 (42-97)% |  | 59 (38-78)% |  | 48 (27-69)% | 0.34 |
| 2-year |  | 56 (19-90)% |  | 59 (38-78)% |  | 48 (27-69)% | 0.74 |
| 3-year |  | 56 (19-90)% |  | 59 (38-78)% |  | 48 (27-69)% | 0.74 |
| 4-year |  | 56 (19-90)% |  | 48 (28-69)% |  | 48 (27-69)% | 0.93 |

Abbreviations: GVHD=graft-versus-host disease; Prob= probability; CI = confidence interval; N = number; NRM=non-relapse mortality; PFS=progression-free survival
